# BLOOD CELLS PROFILE IN UMBILICAL CORD OF LATE PRETERM AND TERM NEWBORNS

**DOI:** 10.1590/1984-0462/;2019;37;3;00008

**Published:** 2019-06-19

**Authors:** Anna Carolina Boni Rolim, Marley Aparecida Lambert, Juliana Policastro Grassano Borges, Samira Ali Abbas, José Orlando Bordin, Dante Mário Langhi, Akemi Kuroda Chiba, Amélia Miyashiro Nunes dos Santos

**Affiliations:** a Universidade Federal de São Paulo, São Paulo, SP, Brazil.; b Irmandade da Santa Casa de Misericórdia de São Paulo, São Paulo, SP, Brazil.; c Santa Casa de São Paulo, São Paulo, SP, Brazil.

**Keywords:** Infant, newborn, Infant, premature, Blood cell count, Umbilical cord, Recém-nascido, Recém-nascido prematuro, Hemograma completo, Cordão umbilical

## Abstract

**Objective:**

To describe the hematological profile in cord blood of late preterm and term newborns and compare blood indices according to sex, weight for gestational age and type of delivery.

**Methods:**

Cross-sectional study with late preterm and term newborns in a second-level maternity. Multiple gestation, chorioamnionitis, maternal or fetal hemorrhage, suspected congenital infection, 5-minute Apgar <6, congenital malformations, and Rh hemolytic disease were excluded. Percentiles 3, 5,10, 25, 50, 75, 90, 95 and 97 of blood indices were calculated for both groups.

**Results:**

2,662 newborns were included in the sample, 51.1% males, 7.3% late preterms, 7.8% small for gestational age (SGA) and 81.2% adequate for gestational age (AGA). Mean gestational age was 35.6±1.9 and 39.3±1.0 weeks, respectively, for premature and term neonates. The erythrocytes indices and white blood cells increased from 34-36.9 to 37-41.9 weeks. Basophils and platelets remained constant during gestation. Premature neonates presented lower values ​​of all blood cells, except for lymphocytes and eosinophils. SGA neonates presented higher values ​​of hemoglobin, hematocrit and lower values of leukocytes, neutrophils, bands, segmented, eosinophils, monocytes and platelets. Male neonates presented similar values ​​of erythrocytes and hemoglobin and lower leukocytes, neutrophils, segmented and platelets. Neonates delivered by C-section had lower values ​​of red blood cells and platelets. Chronic or gestational hypertension induced lower number of platelets.

**Conclusions:**

Blood cells increased during gestation, except for platelets and basophils. SGA neonates had higher hemoglobin and hematocrit values and lower leukocytes. Number of platelets was smaller in male SGAs, born by C-section and whose mothers had hypertension.

## RESUMO

**Objetivo:**

Descrever o perfil hematológico em sangue de cordão de recém-nascidos pré-termo tardio e a termo e comparar parâmetros hematimétricos segundo sexo, adequação peso idade gestacional e tipo de parto.

**Métodos:**

Estudo transversal com recém-nascidos pré-termo tardio e a termo, em maternidade de nível secundário. Excluíram-se gestação múltipla, corioamnionite, hemorragia materna ou fetal, suspeita de infecção congênita, Apgar no 5^o^ minuto <6, malformações congênitas e doença hemolítica Rh. Calcularam-se os percentis 3, 5, 10, 25, 50, 75, 90, 95 e 97 dos parâmetros hematológicos.

**Resultados:**

Incluíram-se 2.662 recém-nascidos, 51,1% do sexo masculino, 7,3% prematuros tardios, 7,8% pequenos para a idade gestacional e 81,2% adequados. A idade gestacional foi 35,6±1,9 e 39,3±1,0 semanas, respectivamente, nos prematuros e termos. As séries vermelha e branca aumentaram de 34-36,9 para 37-41,9 semanas, exceto basófilos e plaquetas, que permaneceram constantes. Os prematuros apresentaram menores médias nas séries vermelha, plaquetária e branca, com exceção de linfócitos e eosinófilos. Recém-nascidos pequenos para a idade gestacional apresentaram maiores valores de hemoglobina e hematócrito e menores de leucócitos, neutrófilos, bastonetes segmentados, eosinófilos, monócitos e plaquetas. Recém-nascidos masculinos apresentaram taxas semelhantes de hemoglobina e hematócrito e menores de leucócitos, neutrófilos, segmentados e plaquetas. Na cesárea, as células vermelhas e as plaquetas foram menores que no parto vaginal. O número de plaquetas foi menor na hipertensão crônica ou gestacional.

**Conclusões:**

As células sanguíneas aumentaram durante a gestação, exceto plaquetas e basófilos. Recém-nascidos pequenos para a idade gestacional apresentaram maiores taxas de hemoglobina e hematócrito e menores de células brancas. O número de plaquetas foi menor no recém-nascido pequeno para a idade gestacional, masculino, nascido por cesárea e de mãe hipertensa.

## INTRODUCTION

Currently, there are few publications on complete blood cell count from umbilical cord blood.^1^ ^,^ ^2^ Ozurek et al.^1^ have reported higher values of normoblasts, hemoglobin, hematocrit and erythrocytes in cord blood count of newborns small for gestational age (SGA) compared to infants adequate for gestational age (AGA). Among SGAs, 21.9% were reported to have neutropenia and 4.7% presented with neutrophils below 1,500 u/µL on the first day.

Hemoglobin and hematocrit values from cord blood are useful for indications of red blood cell transfusions or, when associated with bilirubin values in the cord, for indication of intravenous immunoglobulin in Rh hemolytic disease.^3^ Platelet count helps to monitor immune thrombocytopenia and preeclampsia.^4^ ^,^ ^5^ Cord leukogram’s reliability to identify risk of infections at birth is questionable.^6^ ^,^ ^7^ Nevertheless, some authors suggest that leukogram analysis, along with C-reactive protein analysis, may help diagnose bacterial infections of maternal origin.^8^ ^,^ ^9^

Thus, an analysis of umbilical cord blood cell count has the advantage of reducing blood spoliation, keeping the newborn (NB) from being exposed to vascular puncture and risk of infections, besides allowing clinical decision-making upon birth in some situations.^3^ ^,^ ^4^ ^,^ ^10^

In this context, the objective of this study was to describe the hematological profile of late preterm and term newborns by analyzing their cord blood and to compare blood parameters according to gender, adequacy of weight and gestational age, and type of delivery at a maternity in the Metropolitan Region of São Paulo.

## METHOD

This is a cross-sectional study conducted with live births of Hospital São Luiz Gonzaga from July 2007 to February 2009.

It is part of a research project entitled “Evaluation of frequency of neonatal alloimmune neutropenia in Brazilian newborns”, with free and informed consent signed by the legal representatives of NBs included in the original project (project no. 0051/07). The current project was approved by the Research Ethics Committee of *Universidade Federal de São Paulo* (Unifesp) (project no. 1624/08), with the informed consent form of the original project being considered.

Hospital São Luís Gonzaga is a maternity affiliated to Santa Casa de São Paulo, where approximately 2,000 births happen every year. The maternity unit has 30 rooming-in beds, and the neonatal unit has eight intermediate care beds plus six intensive care beds.

Consecutive live births that occurred in the study period were included, and a sample of umbilical cord blood of each newborn was collected to perform complete blood count.

NBs with gestational age <34 weeks or gestational age >42 weeks, multiple gestations, mother with positive serology for human immunodeficiency virus (HIV), syphilis (venereal disease research laboratory - VDRL, and positive treponemal test), positive serology (immunoglobulin G - IgG - and immunoglobulin M - IgM) for toxoplasmosis and cytomegalovirus, signs of chorioamnionitis (premature amniorrexis plus at least two clinical or hematological signs of maternal-fetal infection), previous placental abruption, 5-minute Apgar <6, major congenital malformations, Rh hemolytic disease (direct and indirect positive Coombs), maternal or neonatal hemorrhage, clinical signs of congenital infection or early neonatal sepsis suspicion were excluded.

After birth, 5 mL of umbilical vein blood was collected in a tube containing ethylenediaminetetraacetic acid anticoagulant (EDTA) for complete blood count and analysis of hematological parameters of neonates. Blood was collected by the nursing team, along with the collection for blood typing of newborns, up to two minutes after placenta discharge. Late umbilical cord clamping routine was not being used at the service at the time of the study.

The complete blood count of all neonates was performed routinely at the hospital’s birth laboratory. The device CellDyn 3700-Abbott^®^ was used by automated method with electronic count of red cells, red cell volume measurement, hemoglobinometry, complementary microscopy of blood extensions stained with Romanowsky dye. The parameters measured directly by the apparatus were: number of erythrocytes (u/mL), hemoglobin concentration (g/dL), hematocrit (%), mean corpuscular volume (MCV: fL), and Red Cell Distribution Width (RDW: %). Parameters calculated were: mean corpuscular hemoglobin (MCH: pg) and mean corpuscular hemoglobin concentration (MCHC: mg/dL). In addition, the number of leukocyte (u/mL) and platelet series (u/µL) were evaluated.

The results of the blood cell count were analyzed and presented in percentiles 3, 5, 10, 25, 50, 75, 90, 95 and 97 for both gestational age ranges: 34 to 36.9 weeks (late preterm NB) and 37 to 41.9 weeks (term NB). Percentiles 3 and 97 nearly correspond to respectively, to -2 and +2 Z scores.

In addition, blood parameters were compared between SGA and AGA newborns, male and female, C-section and vaginal delivery, and children of mothers with and without diabetes or arterial hypertension.

Demographic and clinical data of mothers were collected from medical reports described by the obstetrics team, and also based on records of infants with up to 48 hours of life. Birth weight was measured using a Filizola^®^ digital scale, with maximum capacity of 15 kg, minimum of 250 g, and precision level of 5 g.

The gestational age was attributed by the hospital’s neonatology team, which routinely considered the best obstetric estimate based on the date of last menstrual period or ultrasound examination before 14 weeks.^11^ In the absence of such data or in the presence of difference of two weeks or more between obstetric and pediatric evaluation, the evaluation by the pediatrician was considered.^12^

Adequacy of weight for gestational age was assessed by the Intergrowth curve,^11^ considering AGA the NBs with birth weight between the 10^th^ and 90^th^ percentiles; and SGA the NBs with birth weight below the 10^th^ percentile; NBs large for gestational age (LAG) were those weighing above the 90^th^ percentile.

The numerical variables were evaluated by the Kolmogorov-Smirnov test for the Gauss distribution and compared by the Student’s t-test or the Mann-Whitney’s test for variables with normal distribution (expressed as mean and standard deviation) or asymmetric distribution (expressed in median and minimum-maximum values), respectively. Categorical variables were described as number and percentage and compared by the chi-square or Fisher’s exact test.

Statistical analyses were performed with the aid of the Statistical Package for Social Sciences (SPSS) version 17 (SPSS Inc., Chicago, IL, USA). Statistical significance level was set at p<0.05.

## RESULTS

**Figure 1:**
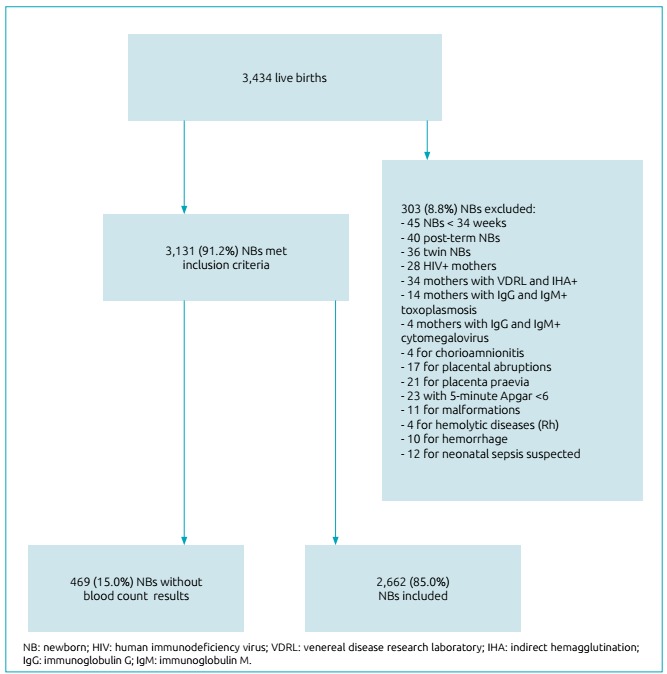
Flowchart of patients selected.

During the study period, there were 3,434 live births at the studied hospital. Of these, 303 (8.8%) were excluded. Of these, 45 (1.3%) had been born before 34 weeks of gestation; 40 (1.2%) had gestational age >42 weeks; 36 (1.0%) were twins; 28 (0.8%) had mothers with positive serology for HIV; 34 (1.0%) had positive VDRL and indirect hemagglutination (IHA); 14 (0.4%) had positive IgG and IgM for toxoplasmosis; 4 (0.1%) had positive IgG and IgM for cytomegalovirus; 4 (0.1%) pregnant women presented with clinical chorioamnionitis; 17 (0.5%) had placental abruption; 21 (0.6%) had placenta praevia; 23 (0.7%) had 5-minute Apgar <6; 11 (0.3%) newborns had malformations; 4 (0.1%) had Rh hemolytic disease; 10 (0.3%) had bleeding at birth; and 12 (0.3%) had early sepsis suspicion (Figure 1).

Among 3,131 newborns that met the inclusion criteria, 469 (15.0%) neonates did not have blood cell count results due to blood clotting, material loss or parents’ refusal. Thus, 2,662 (85.0%) NBs who met the inclusion criteria were included in the study (Figure 1).

Maternal ages (26.5±7.7 versus 26.0±6.5 years, p=0.391), number of pregnancies (2.7±1.7 versus 2.5±1.7, p=0.190) and number of previous deliveries (1.5±1.6 versus 1.3±1.6, p=0.177) were similar between late preterm and term NBs, respectively. Chronic hypertension (7.6 versus 3.6%, p=0.007), gestational hypertensive disease (4.3 versus 2.1%, p=0.045), and urinary tract infection (5.5 versus 2,6%, p=0.022) were more frequent in mothers of late preterm. Diabetes (1.1 versus 0.4%, p=0.204) and gestational diabetes (1.7 versus 1.2%, p=0.370) were similar in both groups. 34.2% of preterm versus 29.2% of term neonates were delivered by C-section (p=0.139).

Among 2,662 NBs, 1,359 (51.1%) were males, 193 (7.3%) were late preterm, 208 (7.8%) were SGA, 293 (11.0%) were LGA, and 2,161 (81.2%) were AGA. There were no differences in the proportions of male and SGA neonates between groups.

The mean gestational age of late preterm newborns was 35.6±1.9 weeks, versus 39.3±1.0 weeks for term newborns. Birth weight was 2,580±457 and 3,271±437 g in late preterm and term neonates, respectively. Preterm NBs had lower 1st-minute (8.0±1.2 versus 8.2±1.2, p=0.011) and 5th-minute Apgar score (9.1±0.7 versus 9.3±0, 7, p<0.001), and the occurrence of 1st-minute Apgar from 0-3 was similar in both groups (1.6 versus 0%, p=0.079).

Among 193 late preterm infants, neonatal complications in the first 48 hours were respiratory distress syndrome (0.5%), transient tachypnea (9.3%), pulmonary hypertension (1.6%), persistent ductus arteriosus (0.5%), and no late preterm infant had a diagnosis of peri-intraventricular hemorrhage.

The values of percentiles 3, 5, 10, 25, 50, 75, 90, 95 and 97 for the number of erythrocytes, hemoglobin and hematocrit rate, MCV, MCH, MCHC, RDW, number of leukocytes, neutrophils, lymphocytes, eosinophils, basophils and platelets are shown in Tables 1 and 2.

**Table 1:** Percentiles of blood parameters in cord blood in late preterm (34-36 6/7 weeks) and term neonates (37-41 6/7 weeks).

	N	P3	P5	P10	P25	P50	P75	P90	P95	P97
Red blood cells (u/mL)										
Late preterm	193	3,239,200	3,391,000	3,694,000	4,020,000	4,370,000	4,770,000	5,088,000	5,223,000	5,424,400
Term	2,469	3,510,000	3,660,000	3,890,000	4,200,000	4,550,000	4,890,000	5,190,000	5,400,000	5,520,000
Hb (g/dL)										
Late preterm	193	11.3	11.8	13.0	14.3	15.5	17.0	18.0	18.5	18.5
Term	2.469	12.0	13.0	13.0	14.0	16.0	17.0	18.0	19.0	19.0
Hct (%)										
Late preterm	193	34.4	36.5	39.1	42.0	46.0	51.0	55.0	56.0	56.5
Term	2.469	38.0	39.0	41.0	44.0	48.0	52.0	55.0	57.0	58.0
MCV (fL)										
Late preterm	193	97.5	98.1	100.2	104.5	108.0	112.4	115.4	118.1	118.8
Term	2.469	94.0	96.0	98.0	102.0	106.0	110.0	113.0	115.0	117.0
MCHC (g/dL)										
Late preterm	193	30.5	30.8	31.3	32.1	33.1	34.1	35.0	35.2	35.5
Term	2.469	30.0	30.0	31.0	32.0	33.0	34.0	35.0	35.0	35.0
RDW (%)										
Late preterm	193	14.9	15.1	15.2	15.9	16.8	18.1	19.6	20.4	21.2
Term	2.469	14.0	14.0	15.0	15.0	16.0	18.0	19.0	20.0	20.0
Platelets (u/mL)										
Late preterm	193	129,000	141,100	201,200	243,750	299,000	338,000	392,600	420,450	435,540
Term	2,469	128,120	149,600	190,100	245,000	297,000	343,000	394,800	428,000	451,440

u/mL: units per microliter; Hb: hemoglobin; Hct: hematocrit; MCV: mean corpuscular volume, fL: fentoliter; MCHC: mean corpuscular hemoglobin concentration; RDW: red cell distribution width.

**Table 2:** Percentiles of blood parameters in cord blood in late preterm (34-36 6/7 weeks) and term neonates (37-41 6/7 weeks).

	n	P3	P5	P10	P25	P50	P75	P90	P95	P97
Leukocytes (u/mL)										
Late preterm	193	6,164	6,670	7,500	9,000	11,400	14,600	18,900	21,380	22,072
Term	2,469	7,900	8,500	9,600	11,350	13,900	17,000	20,500	23,450	25,700
Neutrophils (u/mL)										
Late preterm	193	1,751	2,307	2,608	3,758	5,610	8,349	10,930	12,926	14,239
Term	2,469	3,430	3,900	4,602	6,030	7,866	10,037	12,734	14,769	16,497
Bands (u/mL)										
Late preterm	192	0	0	0	0	132	340	612	994	1,218
Term	2,456	0	0	0	0	206	440	789	1,081	1,313
Segmented (u/mL)										
Late preterm	193	1,751	2,222	2,497	3,579	5,328	7,911	10,204	11,898	13,540
Term	2,469	3,152	3,702	4,400	5,828	7,534	9,593	12,028	13,973	15,442
Lymphocytes (u/mL)										
Late preterm	193	2,276	2,433	2,937	3,696	4,560	5,671	7,533	8,224	9,938
Term	2,458	2,129	2,400	2,884	3,636	4,718	6,034	7,720	8,696	9,646
Monocytes (u/mL)										
Late preterm	193	80	96	150	314	570	938	1,327	1,670	1,827
Term	2,461	112	137	216	407	696	1,074	1,505	1,892	2,171
Eosinophils (u/mL)										
Late preterm	193	0	0	0	125	230	403	621	855	1,131
Term	2,469	0	0	0	110	232	424	689	888	1,040
Basophils (u/mL)										
Late preterm	192	0	0	0	0	0	0	10	80	107
Term	2,465	0	0	0	0	0	0	63	132	163

u/mL: units per microliter.

Comparison of blood parameters between late preterm and term NBs showed that mean erythrocytes (u/mL) (4,3642,435±563,422 versus 4,545,504±525,608; p<0.001), hemoglobin (g/dL) (15.2±2.1 versus 16.0±1.9, p=0.011) and hematocrit (%) (47.0±6.4 versus 48.3±5.6, p=0.006) increased from 34-36.9 to 37-41.9 weeks of gestation. MCV (fl) values (108.1±5.9 versus 106.5±5.9, p<0.001) and MCH (pg) (35.8±2.7 versus 35.3±2.6; p=0.009) decreased in the course of gestation, and mean values of MCHC (g/dL) (33.1±1.4 versus 33.1±1.5, p=0.913) and RDW (17.1±1.6 *versus* 17.1±1.7; p=0.928) were similar in both ranges of gestational age.

The mean number of leukocytes (u/mL) (12,211±4,328 versus 14,608±4,725, p<0.001), and neutrophils (u/mL) (6,230±3,347 versus 8,365±3,374, p <0,001) and segmented (u/mL) (5,907±3,074 versus 7,964±3,182; p<0.001) increased from late preterm to term NBs. The number of lymphocytes (u/mL) was similar in both gestational age ranges (4,965±2,016 versus 5,042±2,063, p=0,615). The median (minimum-maximum) number of bands (u/mL) - 132 (0-2,340) versus 206 (0-3,904), p=0.001 - and monocytes (u/mL) - 570 (0-3,300) versus 696 (0-4228), p=0.003 - increased from 34-36.9 to 37-41.9 weeks of gestation. However, the number of eosinophils - 230 (0-2,180) versus 232 (0-2640), p=0.877 - and basophils - 0 (0-272) versus 0 (0-638), p=0.817, respectively, were similar for late preterm and term newborns.

Likewise, there was no difference between the mean number of platelets (293,115±77,013 versus 293,552±83,777; p=0,944) for late preterm and term infants.

**Figure 2:**
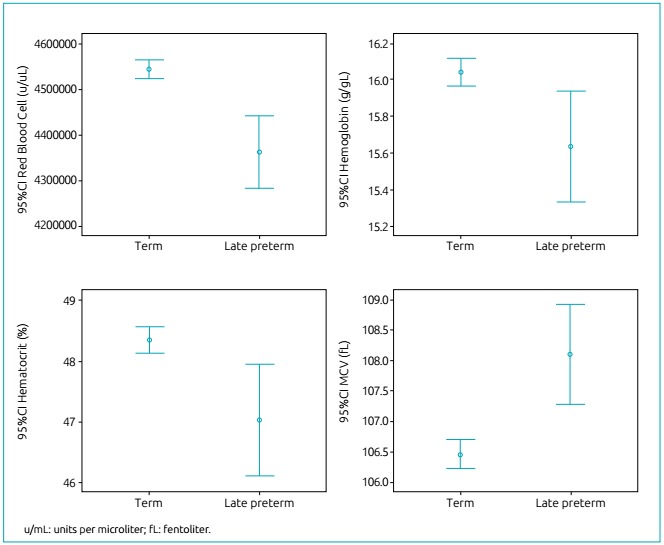
Distribution of erythrocyte indices (mean and 95% confidence interval) in term and late preterm newborns.

**Figure 3:**
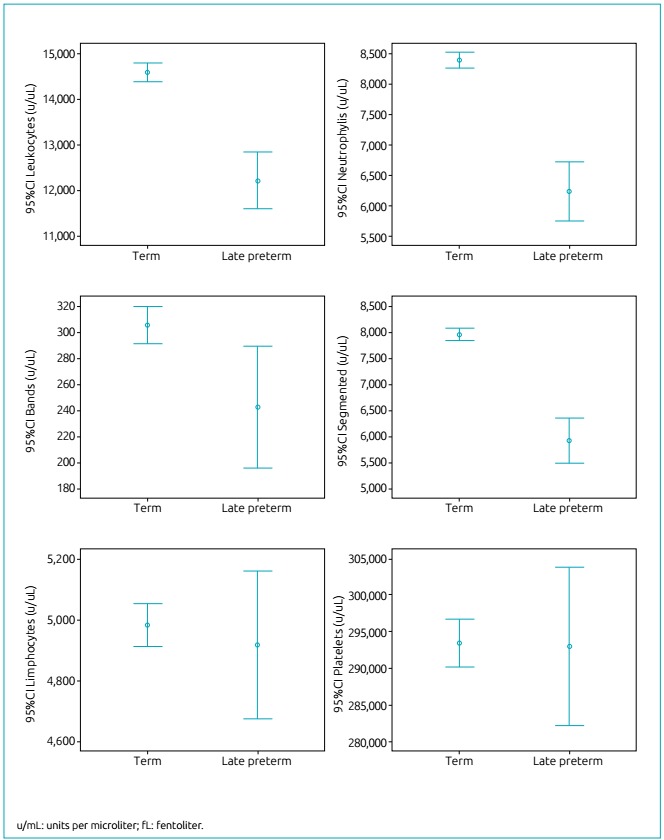
Distribution of white blood cell series and platelet (mean and 95% confidence interval) in term and late preterm newborns.

The distribution of blood values was shown to have greater amplitude for all parameters of the hematological profile in late preterm compared to term infants, as evidenced by the widest 95% confidence interval (95%CI) (Figures 2 and 3).

Comparisons of blood indices between SGA and AGA newborns are shown in Table 3. When comparing LGA and AGA newborns, hemoglobin (g/dL) (15.8±1.8 versus 16.0±1.9; (p<0.05) and hematocrit values were similar (47.8±5.3 versus 48.1±5.6, p=0.254), leukocytes (u/mL) were higher (15,157±4,657 versus 14,457±4,796, p=0.019), and no differences were found for the rest of the white-cell and platelet series.

**Table 3:** Blood parameters in umbilical cord blood of newborns, according to weight adequacy for gestational age.

	SGA (n=208)	AGA (n=2.161)	p-value
RBC (u/mL)	4,607,163±528,849	4,533,319±526,634	0.054*
Hb (g/dL)	16.4±1.9	16.0±2.0	0.003*
Hct (%)	49.7±5.9	48.2±5.6	<0.001*
MCV (fL)	107.9±6.4	106.4±5.9	<0.001*
MCH (pg)	35.6±2.6	35.3±2.6	0.067*
MCHC (g/dL)	33.1±1.5	31.2±1.5	0.293*
RDW (%)	17.0±1.6	17.0±1.6	0.961*
Leukocytes (u/mL)	13,176±4,045	14,457±4,796	<0.001*
Neutrophils (u/mL)	7,365±2,998	8,244±3,450	0.001*
Bands (u/mL)	135 (0-390)	193 (0-420)	0.024^#^
Segmented (u/mL)	7,114±2,843	7,830±3,242	0.001*
Lymphocytes (u/mL)	4,827±1,975	5,027±2,087	0.185*
Eosinophils (u/mL)	196 (87-342)	230 (110-424)	0.017^#^
Monocytes (u/mL)	627 (343-1,008)	676 (396-1,062)	0.048^#^
Basophils (u/mL)	0 (0-0)	0 (0-0)	0.561^#^
Platelets (u/mL)	281,747±89,987	294,262±83,707	0.033*

SGA: small for gestational age; AGA: adequate for gestational age; RBC: red blood cells; u/mL: units per microliter; Hb: hemoglobin; g/dL: gram per deciliter; Hct: hematocrit; MCV: mean corpuscular volume; fL: fentoliter; MCH: mean corpuscular hemoglobin; pg: picogram; MCHC: mean corpuscular hemoglobin concentration; RDW: red cell distribution width; *p-value: t-test, expressed as mean ± standard deviation; #p-value, Mann-Whitney’s test expressed in median (q1-q3).

Compared to females, male NBs presented similar hemoglobin (g/dL) (16.1±1.9 versus 15.9±2.0, p=0.140) and hematocrit values (%) (48.4±5.8 *versus* 48.1±5.5; p=0.267), and lower leukocytes (u/mL) (14,183±4,710 versus 14,692±4,754, p=0.006), neutrophils (u/mL) (7,935±3,318 *versus* 8,451±3,527; p<0.001), segmented (u/mL) (7,560±3,133 *versus* 8,080±3,287; p<0.001) and platelets (u/mL) (288,210±82,487 versus 299,056±83,840, p=0.001).

Children born by C-section had a lower erythrocytes (u/mL) (4,464,407±511,983 versus 4,559,975±536,010, p <0.001), lower hemoglobin (g/dL) (15.8±1.9 *versus* 16.1±2.0; p=0.002), lower hematocrit (%) (47.8±5.7 versus 48.4±5.6, p=008) and lower number of platelets (u/mL) (280,637±81,264 versus 298,840±83,590, p<0.001), with no differences in the white-cell series.

NBs from mothers with diabetes mellitus or gestational diabetes presented red blood cell parameters similar to non-diabetic ones. On the other hand, infants from mothers with hypertension, were shown to be different for platelet values (u/mL) (274,220±92,209 versus 294,400±82,899, p=0,018), as well as NBs of mothers with gestational hypertensive disease (261,118±81,390 versus 294,568±83,096, p=0.002), compared with infants of mothers without such diseases.

## DISCUSSION

We could see a progressive increase in blood cell count during pregnancy from 34 weeks of gestation, with the exception of basophils and platelets, which remained nearly constant from 34 to 41.9 weeks of gestation. The comparison between the late preterm and term NBs showed lower mean number of red blood cells, platelets and white series, except for lymphocytes and eosinophils in former neonates. SGA infants, when compared to AGA NBs, had higher hemoglobin and hematocrit values, but lower white cells and platelets. Male NBs had similar erythrocyte values ​​and a lower leukocyte, neutrophils, segmented and platelets levels. Children born by C-section presented with lower values for red cells and platelets. Infants from mothers with diabetes mellitus or gestational diabetes had similar hematimetric values ​​compared to nondiabetic mothers. NBs of mothers with chronic or gestational hypertension had lower platelets levels compared to mothers without hypertension.

The hematological profile found in our study was similar to what other authors have reported, with slight variations in the number of cells.^13-17^ In a retrospective study with blood collected in the first six hours of life in NBs born with 22-40 weeks of gestation, the hematocrit increased by 0.64% and hemoglobin by 0.21 g/dL for each week of gestation.^16^ Another study showed that in NBs of 22 to 42 weeks of gestational age, the percentile 5 for hemoglobin level ranged from 12 to 14 g/dL, and the 95th percentile, from 18 to 22 g/dL.^15^ Lee et al.^17^ described no difference in the number of leukocytes, neutrophils, lymphocytes, monocytes, eosinophils and basophils between different gestational ages in samples collected from umbilical cord of healthy newborns. Christensen et al.^18^ reported that the mean values of eosinophils and monocytes on the day of birth increased linearly between 22 and 42 weeks of gestational age.

In our study, MVC, MCH and RDW showed little variation in pregnancy, similarly to other studies.^14^ ^,^ ^15^ MVC estimates the mean red cell size and has a direct correlation with MCH. High levels of MCHC may indicate that erythrocytes are spherical, assuming that MCHC increases in the presence of spherocytosis, but it is not known whether this index helps to identify infants with hemolysis from ABO incompatibility.^19^

From the clinical point of view, although the blood parameters were reported to have small differences when each parameter related to red, white and platelet series were analyzed, 95%CI was much higher among late preterm NBs than among term infants, which shows the difficulty of interpreting blood count results in preterm infants.

Compared to AGA, SGA NBs had higher hemoglobin and hematocrit levels, lower white cells - except lymphocytes and basophils - and lower number of platelets. Studies suggest that increased erythropoiesis and reduced white cells are associated with prolonged exposure to hypoxia.^20^ ^,^ ^21^ It is suggested that frequent placental infarctions with platelet consumption may lead to thrombocytopenia and polycythemia, and that preeclampsia and chronic hypertension may play a role in the genesis of thrombocytopenia.^5^

Male NBs had similar erythrocyte parameters and lower leukocyte and platelet values, compared to the females. Data in the literature are controversial, but studies conducted with large samples did not report differences between the genders.^15^ ^,^ ^22^ Schmutz et al.^23^ reported an average of 2,000 more neutrophils in female NBs.

NBs delivered by vaginal birth presented higher red cell and platelet numbers compared to those born by C-section, possibly due to the higher body water content in cesarean births.^24^ It is possible that the transfer of water from the intravascular to the extravascular system is triggered by labor and that the start of this process is intrauterine.^24^ A meta-analysis showed mean difference in hematocrit levels of -2.91% (95%CI -4.16- -1.6) and of -0.51 (95%CI -0,79- -0,27) in hemoglobin levels between cesarean and vaginal delivery.^25^ We found no differences in the white cell series as related to the type of delivery, but Schmutz et al. referred that neonates born after labor had a greater number of neutrophils in the first 72 hours than those born by elective cesarean section.^23^ The reason is unknown, with some authors attributing a beneficial immunological effect for the stress of labor,^26^ while a recent review has shown controversial results as for the influence of type of delivery on oxidative processes in newborns.^27^ The increase in platelets would be explained by the increase of thrombopoietin and cortisol levels in vaginal delivery.^28^ ^,^ ^29^

Late preterm NBs presented greater variability of blood parameters, in contrast to term NBs. This may represent a limitation of this research when it comes to sample size.

Nevertheless, this study has internal validity, especially for term newborns, and shows the possibility to collect blood from umbilical cord for blood cell count. Henry et al. reported that one of the factors that contributed to reduce the number of transfusions was having initial laboratory tests performed in cord blood samples.^10^ Another multicenter study showed that collecting first test samples from umbilical cord was a feasible technique and that NBs tested for cord blood had an increase in hemoglobin in up to 12-24 hours and received fewer vasoactive drugs and transfusions compared to those who had peripheral blood tested.^30^

Conclusion is that the number of blood cells increased during gestation, except for basophils and platelets. Hemoglobin and hematocrit rates were higher and white cells and platelets were lower in SGA neonates. Among males, there was no difference in the red-cell series, but a lower number of white cells and platelets was reported in comparison with females. Infants born by C-section had lower rates of red-cells and platelets. NBs of mothers with hypertension or gestational hypertensive disease also presented lower platelets.
